# The lncRNA HOTAIR: a pleiotropic regulator of epithelial cell plasticity

**DOI:** 10.1186/s13046-023-02725-x

**Published:** 2023-06-13

**Authors:** Laura Amicone, Alessandra Marchetti, Carla Cicchini

**Affiliations:** Istituto Pasteur Italia-Fondazione Cenci Bolognetti, Dipartimento di Medicina Molecolare, Sapienza University of Rome, Viale Regina Elena 324, Rome, 00161 Italy

**Keywords:** Epithelial-to-mesenchymal transition (EMT), Epithelial tumor progression, HOTAIR, Metastasis, Long non-coding RNAs

## Abstract

The epithelial-to-mesenchymal transition (EMT) is a trans-differentiation process that endows epithelial cells with mesenchymal properties, including motility and invasion capacity; therefore, its aberrant reactivation in cancerous cells represents a critical step to gain a metastatic phenotype. The EMT is a dynamic program of cell plasticity; many partial EMT states can be, indeed, encountered and the full inverse mesenchymal-to-epithelial transition (MET) appears fundamental to colonize distant secondary sites. The EMT/MET dynamics is granted by a fine modulation of gene expression in response to intrinsic and extrinsic signals. In this complex scenario, long non-coding RNAs (lncRNAs) emerged as critical players. This review specifically focuses on the lncRNA HOTAIR, as a master regulator of epithelial cell plasticity and EMT in tumors. Molecular mechanisms controlling its expression in differentiated as well as trans-differentiated epithelial cells are highlighted here. Moreover, current knowledge about HOTAIR pleiotropic functions in regulation of both gene expression and protein activities are described. Furthermore, the relevance of the specific HOTAIR targeting and the current challenges of exploiting this lncRNA for therapeutic approaches to counteract the EMT are discussed.

## Background

Differentiated cells can respond to microenvironment cues, dramatically and dynamically changing the organization of their components and their core gene expression. Particularly, the plasticity of epithelial cells is guaranteed by the ability to undergo the Epithelial-to-Mesenchymal Transition (EMT), a trans-differentiation process that physiologically ensures mesodermal formation during gastrulation, neural crest delamination, heart development, wound healing and tissue regeneration. Notably, EMT can be responsible for and/or worsen pathological situations, such as fibrosis of epithelial tissues and progression of epithelial tumors [1–3]. EMT involves a profound reorganization of cytoskeleton together with the gradual loss of cell-cell and cell-extracellular matrix adhesions and cell polarity and, finally, with the acquisition of a mesenchymal phenotype. This is featured by increased motility, resistance to apoptosis and invasiveness. In tumor progression, some transformed cells by undergoing the EMT acquire the ability to spread and colonize distant sites, causing metastasis. Moreover, EMT traits in tumoral cells include stem cell-like features, increased tumorigenicity, metabolic reprogramming, therapy resistance and immune evasion, ensuring a pro-survival phenotype in stress conditions [1–3]. Therefore, therapeutic targeting of EMT is a current challenge for translational medicine to control multifaceted aspects of epithelial cancer progression.

Several transcriptional factors have been enrolled as masters of EMT (EMT Transcriptional Factors, EMT-TFs), able to trigger and orchestrate the trans-differentiation process, starting from the direct repression of the epithelial marker E-cadherin. These include the SNAIL family members SNAI1 (SNAIL) and SNAI2 (SLUG), the TWIST factors TWIST1 (TWIST) and TWIST2, and the homeobox factors ZEB1 and ZEB2 [4–6]. However, the epithelial plasticity depends on a fine-balanced network of transcriptional and post-transcriptional regulations of gene expression, on which a plethora of cell-extrinsic stimuli conveys. Particularly in the tumor niche, the coexistence of different cell types (tumor cells, endothelial cells, tumor-associated fibroblasts, immune cells and others), together with extracellular matrix (ECM) components, controls the production of pro- and anti-EMT factors, whose balance results in several intermediate states that transitional cells can acquire and maintain much or less stably [7]. Thus, many situations of partial and dynamic EMT can be encountered, as well as the reversion of the EMT process in the Mesenchymal-to-Epithelial Transition (MET) [8, 9].

The EMT in tumor progression is mainly associated with deregulation of different signaling pathways and with genetic and epigenetic modifications influencing gene expression, with an important role played by different types of aberrantly expressed and functioning noncoding RNAs (ncRNAs), including long noncoding RNAs (lncRNAs) [10].

LncRNAs are molecules longer than 200 nucleotides, derived from Polymerase II transcripts that, as well as the transcripts coding proteins, include an exon-intron structure and are post-transcriptionally processed. These ncRNAs are important regulators of gene expression, localizing in the nucleus or in the cytoplasm and playing multiple functions in the chromatin context or in the regulation of other RNAs and proteins [11, 12].

LncRNAs emerged as key regulators of EMT, being mainly involved in the recruitment of activating or repressive transcriptional complexes on promoters and other regulatory regions of genes, or in the control of expression and/or function of other regulatory RNAs and proteins [13].

Here, we will focus on the HOX Transcript Antisense Intergenic RNA (HOTAIR), a lncRNA that the intense investigation of the last few years has characterized as a master regulator of epithelial cell plasticity and EMT, particularly in the progression of epithelial tumors. By a systematic review of recent literature, we will report the current understanding of its regulation and function, specifically in the EMT context, highlighting its pleiotropic mechanism of action. Furthermore, the potential targeting of HOTAIR in therapeutic approaches will be discussed.

## The lncRNA HOTAIR

### The regulation of HOTAIR expression

**Fig. 1:**
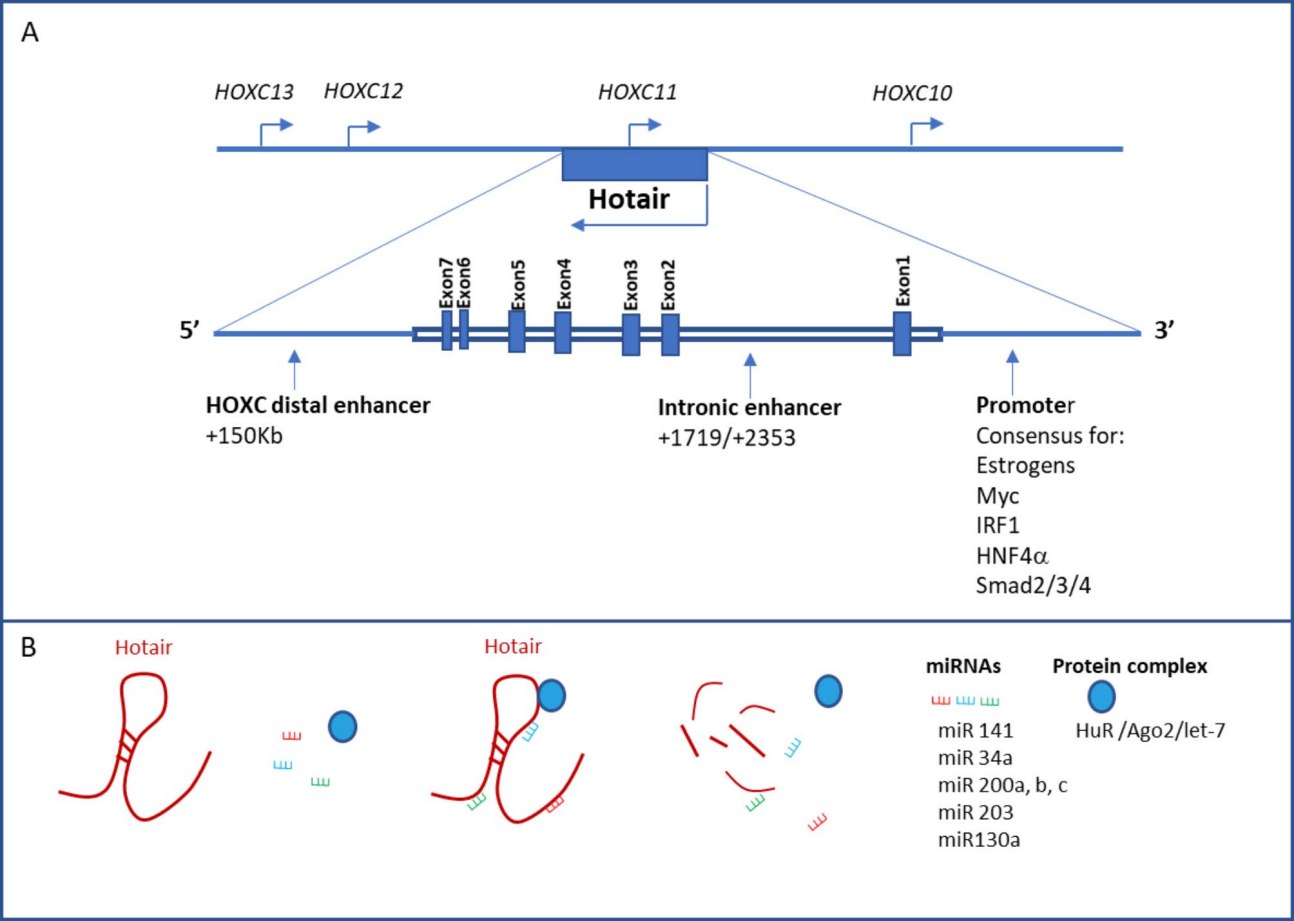
Scheme showing the regulation of HOTAIR expression. **(A)** The antisense transcription of the HOTAIR gene within the HOXC cluster genes can be controlled by a downstream distal enhancer (HOXC distal enhancer) and an intronic enhancer. The functionally characterized consensus sequences in the promoter region upstream the TSS are listed. **(B)** HOTAIR levels are post-transcriptionally controlled through the negative regulation by different miRNAs and through the HuR/Ago/let-7-mediated degradation

HOTAIR is a spliced and poly-adenylated lncRNA, that arises from transcription of the antisense strand of the mammalian HOXC (Homeobox Transcription Factor C) locus [14]. Despite a large body of evidence highlighting the aberrant HOTAIR expression in the progression of different cancers (e.g. breast [15], colorectal [16], nasopharyngeal [17], liver [18], gallbladder [19], gastrointestinal [20] and pancreatic [21] cancers), the mechanisms controlling its transcription are still only partially unveiled. The up to date known elements that are involved in the transcriptional regulation of HOTAIR are depicted in Fig. 1A and described below.

Two enhancer regions have been reported to control HOTAIR overexpression in different cancer cells. Firstly, an intronic enhancer was described by Zhang and Colleagues [22]. These Authors investigated the association between selected haplotype-tagging SNPs in the HOTAIR locus and the susceptibility to esophageal squamous cell carcinoma, in the hypothesis that the genetic variants could affect the expression of the lncRNA gene as well as the functionality of the transcript. Specifically, they found the SNP rs920778 in the intron 2 of HOTAIR gene significantly associated with increased risk of carcinoma occurrence. Notably, the analysis of the H3K4me1 and H3K4me3 modifications, as well as the data of transcriptional activity obtained through luciferase assays by using different gene constructs, identified a putative enhancer in the same intronic region (between + 1719 bp and + 2353 bp from the transcriptional start site) whose activity can be influenced by the specific SNP [22]. Furthermore, by investigating chromatin modifications and long-range interactions in breast cancer cells, Milevskiy and coworkers identified a HOXC cis-regulatory element, termed HOXC Distal Enhancer (HDE), approximately 150 kb downstream of the HOTAIR transcription start site [23]. The interaction between HDE and the promoter region of HOTAIR was proven by chromosome conformation capture (3C) and the HDE function in increasing HOTAIR promoter activity was demonstrated by luciferase reporter assays. Furthermore, this distal enhancer was found dependent on forkhead box (FOX) proteins [23].

Proximal HOTAIR promoter sequences have also been investigated, unveiling sites of binding for different regulators with a role in the EMT process. Bhan and Colleagues [24], unveiled multiple functional GGTCA estrogen response elements (EREs) within 2000 nt upstream of the HOTAIR gene transcription start site and, by using different constructs in luciferase assays, demonstrated their functionality in controlling the lncRNA expression. Moreover, they found that HOTAIR expression in breast cancer cells can be induced by estradiol. The estradiol-dependent activation of HOTAIR expression requires both receptors ERα and ERβ and the recruitment on the HOTAIR promoter of co-regulators such as mixed lineage leukemia (MLL), histone methyltransferases 1 and 3 and histone acetyltransferases CREB-binding protein (CBP)/p300 [24]. Interestingly, estradiol (E2) can promote EMT of breast cancer cells [25], as well as gastric cancer cells, where the induction of trans-differentiation process *via* HOTAIR has been demonstrated [26].

By means of a computational screening and successive validation by luciferase reporter assay, chromatin immunoprecipitation (ChIP) and electrophoretic mobility shift assay (EMSA), Ma and Colleagues identified c-Myc as an activator of HOTAIR transcription, by direct interaction with its target response element in HOTAIR promoter. HOTAIR gene activation, in turn, promotes gallbladder cancer malignancy [19].

Furthermore, by similar approaches Yang G. et al. demonstrated that HOTAIR can be negatively regulated by Interferon Regulatory Factor-1 (IRF1), a transcription factor known to act as tumor suppressor and with a key role in host defense, proliferation, apoptosis, immune and DNA damage responses [27]. Specifically, it has been shown in several cancer cell lines that osteopontin, a secreted protein whose expression has been coupled to tumor progression and metastasis, induces HOTAIR expression inhibiting the expression of IRF1 *via* PI3K/AKT pathway [28].

A key regulator of HOTAIR expression in EMT plasticity is the transforming growth factor β1 (TGF-β1), main inducer of the mesenchymal trans-differentiation both in cultured and in vivo epithelial cells [29]. This pleiotropic cytokine can induce HOTAIR transcription via Smad2/3 as well as by controlling the levels of the orphan nuclear receptor hepatocyte nuclear factor 4-α (HNF4α). This last transcriptional factor is a well-known master regulator of epithelial differentiation [30–32] and a direct repressor of master EMT-TFs, particularly Snail, and mesenchymal genes, thus controlling the maintenance of the epithelial phenotype and triggering the process of MET [33, 34]. Notably, TGF-β1 impairs HNF4α function by inducing post-translational modifications that correlate with the early loss of its DNA binding activity on target gene promoters [34]. Furthermore, TGF-β1 induces Snail that, in turn, directly represses HNF4α expression [35, 36].

A Smad-mediated molecular mechanism of TGF-β1-induced HOTAIR regulation was reported by Ren and Colleagues in breast cancer cells [37]. These Authors demonstrated that the cytokine, mainly secreted by cancer-associated fibroblasts (CAFs) in tumor microenvironment and acting in a paracrine manner, activates HOTAIR gene expression. Specifically, Smad2/3, in complex with Smad4, directly binds HOTAIR promoter sequences between nucleotides − 386 and − 398, -440 and − 452. HOTAIR transcription, in turn, promotes EMT and metastasis of cancer cells [37].

The HNF4α-mediated mechanism of HOTAIR transcriptional regulation was described by Battistelli and Co-workers [38]. The functional role of HNF4α in controlling HOTAIR expression was investigated by means of overexpression, silencing or impairment of protein activity after TGFβ-mediated treatment by using as experimental models (i) hepatocyte cells undergoing the EMT/MET [35, 39, 40], (ii) hepatocyte-specific HNF4α knockout mice [41] and (iii) colon cancer cells mimicking different states of tumor progression [42]. Notably, ChIP analyses provided evidence that, in epithelial cells, HNF4α directly binds to HOTAIR regulatory sequences and this inversely correlates with HOTAIR transcription. Of note, by means of the 3C technique, the Authors demonstrated that HNF4α removes a chromatin loop including the distal enhancer previously characterized by Milevskiy [23] and the proximal promoter of HOTAIR, thus causing a chromatin topological remodeling, non-permissive for the lncRNA expression. On the other hand, in response to TGF-β1, HNF4α is both functionally inactivated and transcriptionally repressed, as previously reported [34, 43] and this allows HOTAIR expression together with the extensive reprogramming of gene expression underlying the EMT process [38]. The induction of HOTAIR expression after TGF-β1 treatment was further described by Padua Alves and Colleagues as a requirement for the EMT induction and the maintenance of stem properties of colon and breast cancer cells [44].

In lung cancer cells, Zhuang et al. identified the type 1 collagen as an inducer of expression of HOTAIR via α2β1 integrin. Even if the cellular signaling pathway induced by collagen requires a deeper investigation, this result appears interesting since it confers to an extracellular matrix component, enriched in the tumor microenvironment, the capacity to trigger the transcription of this tumor-promoting lncRNA [45].

With respect to possible epigenetic mechanisms controlling HOTAIR transcription, Lu et al. [46] investigated in primary breast cancers the methylation status of an intergenic CpG island located between HOXC12 and HOTAIR genes, highlighting a positive correlation between DNA methylation in this region and HOTAIR expression. On the basis of this observation, the Authors suggest that this island may act as an insulator that, in the methylated state, forms a barrier that impedes possible transcriptional interferences with HOTAIR gene expression. Besides Bhan et al., by ChIP analysis in ER-positive breast cancer cells, demonstrated that histone H3K4-trimethylation and histone acetylation are enriched at the ERE regions of HOTAIR promoter in presence of E2, so identified as a HOTAIR inducer. Coherently, MLL histone H3 lysine 4 (H3K4) methyltransferases and CBP/p300 histone acetylase are recruited as E2-coactivators to allow these chromatin modifications permissive for gene expression [24].

The control of HOTAIR expression also includes post-transcriptional mechanisms (Fig. 1B). With respect to the functional role of miRNAs as negative regulators, Wang et al. characterized HOTAIR as a direct target of miR-200c and highlighted that the balance between these two different RNA molecules controls the sensitivity to chemotherapy drugs of ovarian cancer stem cells [47]. The miR-200c–mediated downregulation of HOTAIR in epithelial ovarian cancer cells was also correlated by Yang and colleagues to the decrease of Snail expression, the up-regulation of the E-cadherin, the impairment of the invasion properties of cancer cells and the reduced tumorigenicity in nude mice [48]. In renal carcinoma cells, another miR-200 family member, i.e. miR-141, targets HOTAIR negatively controlling cell proliferation and invasion [49]. The ability of miR-200 family members to suppress HOTAIR is not surprising, taking in account that they are well-known regulators of different EMT-TFs and, consequently, of cell plasticity [50–52]. Conversely, miRs-200 transcription can be negatively controlled by EMT-TFs, such as Snail [52, 53]. Therefore, a reciprocal negative regulation between EMT-TFs and miRs-200 exists and controls the EMT/MET dynamics of epithelial cells. Moreover, in prostate and non-small‐cell lung cancer (NSCLC) cells, HOTAIR can be targeted by miR-34a [54, 55]. Of note, miR-34 is a further well-known negative regulator of EMT and tumorigenesis [56, 57]. Interestingly, miR-34, miR-200 a, b and c are directly upregulated by HNF4α in differentiated hepatocytes and repressed during their trans-differentiation [53]. MiR-203 is a further direct post-transcriptional negative regulator of HOTAIR, as demonstrated in renal cell carcinoma by luciferase reporter assays. Coherently, the effects of HOTAIR suppression on migration and invasion ability of the same cancer cells can be matched by the overexpression of miR-203 [58]. Notably, miR-203 is negatively regulated in EMT and its expression in mesenchymal cells impairs their migratory and invasive capacity in vitro as well as the ability to metastasize in vivo [59]. Besides, in gallbladder cancer, HOTAIR was found targeted by miR-130a [19], a tumor-suppressive miRNA downregulated in the progression of different carcinomas [60, 61]. Interestingly, a reciprocal negative regulation exists between HOTAIR and miR-130a, taking into account the competitive endogenous RNA (ceRNA) activity of the lncRNA (described below as a HOTAIR function in the post-transcriptional regulation of gene expression).

Furthermore, HOTAIR can be bound by the ubiquitous human antigen R (HuR) and this association promotes its decay through the recruitment of the Ago2/let-7 complex [62]. Notably, the tumor-suppressor let-7 is repressed by the EMT-TF Snail [63].

### The role of HOTAIR in transcriptional regulation

HOTAIR is implicated in transcriptional regulation by organizing molecular platforms that include epigenetic modifiers and can be recruited in specific loci of the genome to alter the chromatin state. In the paper by Rinn and Colleagues [14], HOTAIR was identified as a lncRNA able to interact with the Polycomb Repressive Complex 2 (PRC2, comprising the H3 lysine-27 (H3K27) methyltransferase EZH2, SUZ12 and EED), and required for the occupancy of this complex in the HOXD locus. Therefore, HOTAIR was firstly characterized to scaffold PRC2 and to target, by an unclear mechanism, the histone modification activity to specific regions of the genome. PRC2 recruitment, in turn, leads to transcriptional gene silencing by H3K27 trimethylation (H3K27me3). Gupta and Coworkers successively reported that HOTAIR overexpression in epithelial breast cancer cells induces a genome-wide retargeting of PRC2 (and H3K27me3). Notably, the HOTAIR-induced modulation of PRC2-mediated epigenome causes a reprogramming of gene expression that resembles that of embryonic fibroblasts and triggers cancer cell invasiveness [15]. Accordingly, HOTAIR overexpression was correlated to metastasis and poor prognosis of several epithelial tumors [15–21]. Therefore, HOTAIR can act both in *cis* and in *trans* and it is an in vivo predictor of metastasis by causing the specific PRC2 binding to different sites of the genome (in spite to some in vitro evidence indicates that the interaction between PRC2 and long transcripts can be promiscuous [64, 65] and that the HOTAIR-mediated transcriptional repression of a reporter can be PRC2-independent [66]).

**Fig. 2:**
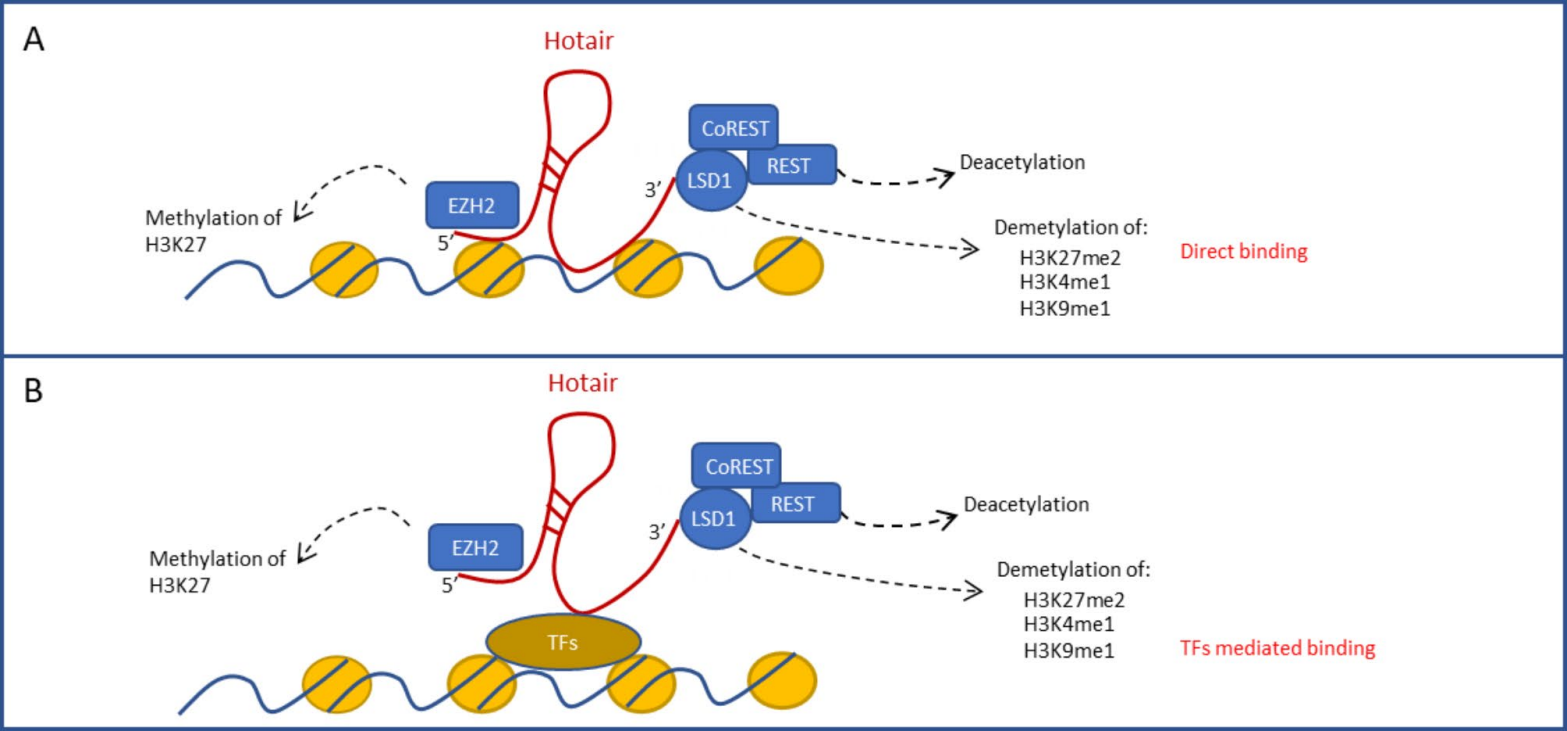
Schemes showing the HOTAIR functions in transcriptional regulation of gene expression. HOTAIR scaffolds distinct repressive histone modification activities (i.e. H3K27 trimethylation by EZH2 component of PRC2 complex, H3K4 and H3K9 demethylation and deacetylation by LSD1/CoREST/REST repressor complex). The targeting of this molecular platform to specific loci of the genome, resulting in the EMT induction, can be dependent **(A)** on the direct formation of triplex helices between the lncRNA and the double stranded DNA target or **(B)** on the formation of tripartite complexes with specific transcriptional factors (e.g. Snail) that confer site-specificity

Mechanistically, in the chromatin environment the lncRNAs can bind to the DNA directly or through the interaction with DNA binding proteins (Fig. 2A and B). Molecular mechanisms of the HOTAIR-mediated targeting of PRC2 to specific loci were investigated in hepatocytes undergoing the EMT by Battistelli and colleagues [67]. These Authors provided evidence that HOTAIR bridges a DNA-binding transcriptional factor, i.e. the EMT-TF Snail, and EZH2, leading to the formation of a Snail/HOTAIR/EZH2 tripartite complex that is instrumental for the execution of the EMT program. Snail, indeed, by means of the direct interaction with HOTAIR, can execute its own repressive activity on epithelial target genes (i.e. HNF4α, HNF1α and E-cadherin), conveying the action of EZH2, main writer of chromatin repressive marks, to these specific sites. RNA pull-down assays after UV crosslinking of cells and purification of complexes in denaturing conditions provided evidence that HOTAIR can scaffold the EMT-TF Snail. RNA immunoprecipitation (RIP) and Co-Immunoprecipitation (Co-IP) experiments established the bridging role of both murine and human HOTAIR in the Snail/HOTAIR/EZH2 tripartite complex. Furthermore, chromatin isolation by RNA purification (ChIRP) and ChIP assays highlighted as the Snail/HOTAIR/EZH2 complex localizes and functions at Snail binding sites on epithelial gene promoters. Notably, HOTAIR was proven epistatic with respect to Snail: Snail binding on target epithelial genes is independent from HOTAIR but its repressive activity depends on HOTAIR/EZH2 recruitment as well as on the related epigenetic modifications [67].

It is conceivable that other transcriptional factors may convey HOTAIR/EZH2 to different sites in the genome and that this mechanism can be accomplished in different cellular processes. Moreover, the reported body of evidence does not rule out the possibility that HOTAIR may be responsible for EZH2 recruitment on specific chromatin contexts by directly associating to DNA sequences. Interestingly, Kalwa and Colleagues [68], on the basis of a computational analysis, identified in HOTAIR sequence five domains predicted to form triple helices in various genomic regions, corresponding to promoters of differentially expressed genes and/or differentially methylated sequences, upon modulation of HOTAIR amount. EMSA assays, using as probes sequences from promoters of genes known to be downregulated by HOTAIR, confirmed the possibility of the interaction in triple helix structure of HOTAIR and DNA, thus enforcing the hypothesis that HOTAIR can be directly recruited on the chromatin to address epigenetic modifiers [68].

Of note, the scaffold ability of HOTAIR is not limited to PRC2 but includes another epigenetic regulator with a key role in EMT reprogramming. By using HOTAIR deletion mutants, Tsai and Colleagues [69] provided evidence that the 3’ domain (nucleotides 1500–2146) of this lncRNA recruits a transcriptional inhibiting complex constituted by the lysine specific demethylase 1 (LSD1) and the deacetylases CoREST/REST, while the 5’ domain (nucleotides 1-300) can bind PRC2. LSD1 can act as a transcriptional corepressor by demethylating histone H3 lysine 4 (H3K4) modifications linked to active transcription [70, 71]. Furthermore, it can also demethylate the repression-associated histone H3 lysine 9 (H3K9) trimethylation, thus exhibiting a role in transcriptional activation [72]. Therefore, HOTAIR appears as a modular bifunctional RNA that scaffolds distinct repressive and activating histone modification activities (i.e. H3K27 methylation by PRC2, H3K4 and H3K9 demethylation by LSD1/CoREST/REST). Coherently with this, Li and collaborators showed that targeted deletion of HOTAIR in mice leads to the loss of H3K27- or gain of H3K4-methylations and derepression of hundred genes [73].

LSD1 activity was previously described to exert a key role in EMT by controlling the reprogramming across the genome of large organized chromatin K9-modifications domains (LOCKs) [74]. Notably, Jarroux et al. highlighted that HOTAIR perturbs the normal LSD1 function at enhancers and promoters, necessary for the maintenance of epithelial identity. Furthermore, the ability of this lncRNA to redistribute LSD1 in the chromatin context is required for the large-scale transcriptional changes that ensure cell migration and the acquisition of mesenchymal traits by epithelial cells. Interestingly, by using deletion mutants, the same Authors highlighted that the HOTAIR-induced epithelial cell migration specifically depends on its LSD1-interacting domain but not on the PRC2-interacting one [75].

### The role of HOTAIR in post-transcriptional regulation

**Fig. 3:**
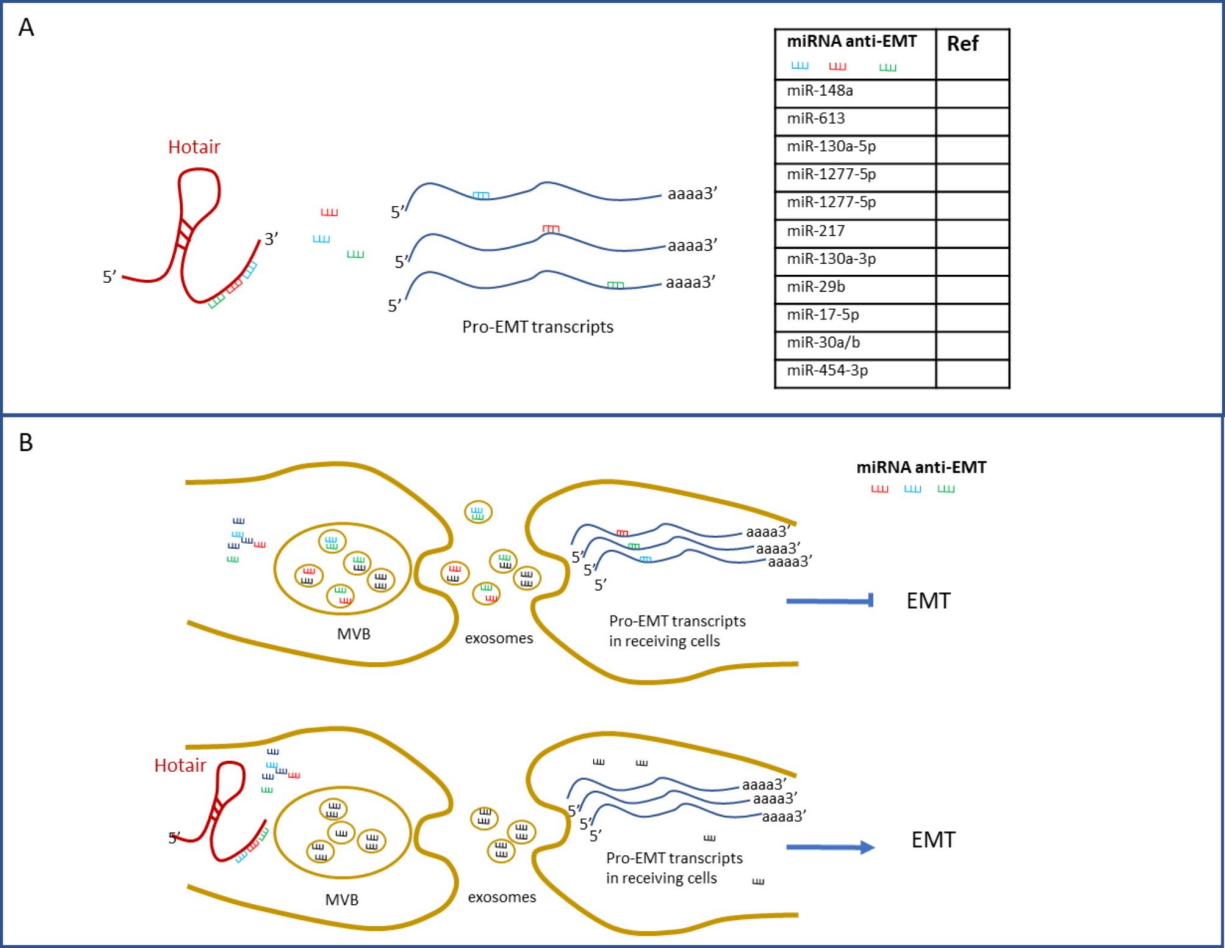
Schemes showing the HOTAIR functions in post-transcriptional regulation of gene expression as a ceRNA in EMT. **(A)** Endogenous (or EV-delivered) HOTAIR sponges specific miRNAs that target pro-EMT transcripts, thus stabilizing them; **(B)** in the intercellular communication, HOTAIR ceRNA activity may control the abundance of anti-EMT miRNAs that are specifically sorted and loaded in exosomes, thus promoting EMT in receiving cells

In line with other known lncRNAs, HOTAIR can act as a ceRNA by “sponging” a number of microRNAs. This function implies (even if it is not limited to that) the ability to regulate EMT and epithelial tumor progression by counteracting the activity of miRNAs with a role in the targeting of key positive players of the trans-differentiation process (Fig. 3A). In esophageal cancer, HOTAIR sponges miR-148a causing a positive modulation of the expression of the EMT-TF Snail2 therefore promoting EMT, invasion and, lastly, metastasis of tumor cells [76]. Interestingly, miR-148 is also known to suppress EMT by regulating further components of EMT-promoting signaling pathways [77–80]. Furthermore, this miRNA is sponged by other oncogenic lncRNAs [81, 82]. In laryngeal squamous cell carcinoma, the HOTAIR-mediated modulation of Snail2 was confirmed as determinant for EMT progression and drug resistance and it was found dependent on the lncRNA-mediated sponge activity on miR-613 [83]. Notably, miR-613 is known to inhibit cancer progression by pleiotropic targeting of multiple signaling pathways [84, 85] and its expression is further controlled by the pro-EMT lncHILAR [86].

ZEB1 is another EMT-TF whose expression is induced by HOTAIR to promote invasiveness and progression of epithelial tumor cells. In esophageal squamous cell carcinoma, Wang and Colleagues reported that HOTAIR promotes the expression of ZEB1 by acting as a ceRNA and negatively regulating miR-130a-5p [87]. Of note, also in gallbladder cancer the oncogenic activity of HOTAIR in promoting invasion and malignancy of tumor cells has been ascribed, at least in part, to its-mediated negative regulation of miR-130a [19]. Notably, this miRNA was also proven to counteract the invasive and metastatic potential of NSCLC cells by interfering with the expression of the transcription factor Runx2 [88]; conversely, its inhibition can be enforced by the lncRNA TYMSOS to promote EMT in thyroid cancer cells [89]. Notably, in colorectal cancer, HOTAIR-mediated modulation of ZEB1 expression was further ascribed to the negative regulation of miR-1277-5p [90]. Of note, the same HOTAIR-miR-1277-5p axis was found to control the deposition in extracellular matrix of collagen type V α1 chain, causal to growth and metastasis of gastric cancer [91]. Moreover, again in gastric cancer, the regulatory role of HOTAIR in promoting EMT was ascribed to the regulation of miR-217 that, in turn, impairs the levels of the GPC5 protein, correlated to proliferation and invasion [92].

HOTAIR further controls the Suv39H1-mediated AKT/mTOR signaling thanks to its sponge activity for miR-130a-3p, thus promoting invasion and metastasis of breast cancer cells [93]. Notably, miR-130a-3p is a negative regulator of other EMT players, e.g. SMAD4 in esophageal squamous cell carcinoma and hepatocarcinoma [94, 95] and Sox4 in glioma [96]. Interestingly, other regulatory RNAs, such as the lncRNA H19 in glioma [96] and the circular RNA hsa_circRNA_102610 in intestinal epithelial cells [97], are known to sponge miR-130a-3p to promote EMT progression.

In cervical cancer, HOTAIR promotes EMT, migration and resistance to chemotherapeutics by sponging miR-29b, thus counteracting the inhibitory role of this miRNA on cancer progression [98]. Notably, mir-29b is a well-known pleiotropic anti-EMT regulator and, therefore, its expression is required to stably maintain the differentiated phenotype of epithelial cells. Interestingly in hepatocytes, HNF4, master factor of MET and epithelial differentiation, inversely regulates the expression of miR-29 family members and of HOTAIR [38, 40].

Moreover, in the paraquat poison-induced fibrosis of lung, HOTAIR sponges miR-17-5p, thus promoting matrix metalloproteinase 2 (MMP2) production and triggering EMT [99]. Notably, miR-17-5p is an anti-metastasis regulator, targeting vimentin [100].

The capacity of lncRNAs to act as sponges for cellular miRNAs also potentially impacts the intercellular communication that is mediated by the extracellular vesicles (EVs) (derived from tumoral cells as well as from other cells of the tumor niche). EVs, indeed, embed different classes of molecules (i.e. proteins, lipids, DNA and RNAs) that have a functional impact on receiving cells and can control their plasticity, exerting a key role for EMT induction and tumor progression toward a malignant phenotype [101, 102]. EVs’ cargo is strictly specific and the sorting process of molecules finely regulated; therefore, in the case of miRNAs that can be specifically secreted *via* EVs, the lncRNA-mediated sponge activity in the cell may conceivably affect the number of miRNAs molecules that effectively result embedded in the EVs (Fig. 3B). Interestingly, in gastric cancer HOTAIR directly binds and induces degradation of miR-30a and −30b and this results in the regulation of both cellular and exosomal expression of these miRNAs. The HOTAIR knockdown in tumor cells, indeed, is associated with more miR-30a and −30b released into the exosomes and determines decreased migration, invasion and proliferation of receiving cells. Conversely, proliferation, migration and invasion are enhanced when tumor cells are co-cultured with exosomes from HOTAIR overexpressing cells [103]. Notably, miRs-30 are pleiotropic key regulators of EMT and, particularly, miR-30 targets the EMT-TF Snail [104].

Furthermore, in line with evidence reported for other lncRNAs, HOTAIR can participate in intercellular communication being itself embedded in EVs, particularly exosomes secreted by different tumor cells, thus representing a circulating marker of disease progression [105–109]. While exosome-delivered HOTAIR may potentially exert all its own different functions in receiving cells, to our knowledge the sole sponge activity has been proposed so far. Cui and Colleagues reported, indeed, that HOTAIR levels increase in recipient laryngeal cancer cells after treatment with exosomes from another line of producing cancer cells; besides, HOTAIR levels correlate with radiosensitivity, because in the same cells this lncRNA can act as a ceRNA for miR-454-3p, thus modulating the transcription factor E2F2 expression [109]. Interestingly, miR-454-3p is known to inhibit several pro-EMT regulators, including the EMT-TF ZEB2 [110] and TGF-β2 [111].

### The role of HOTAIR in post-translational regulation

**Fig. 4:**
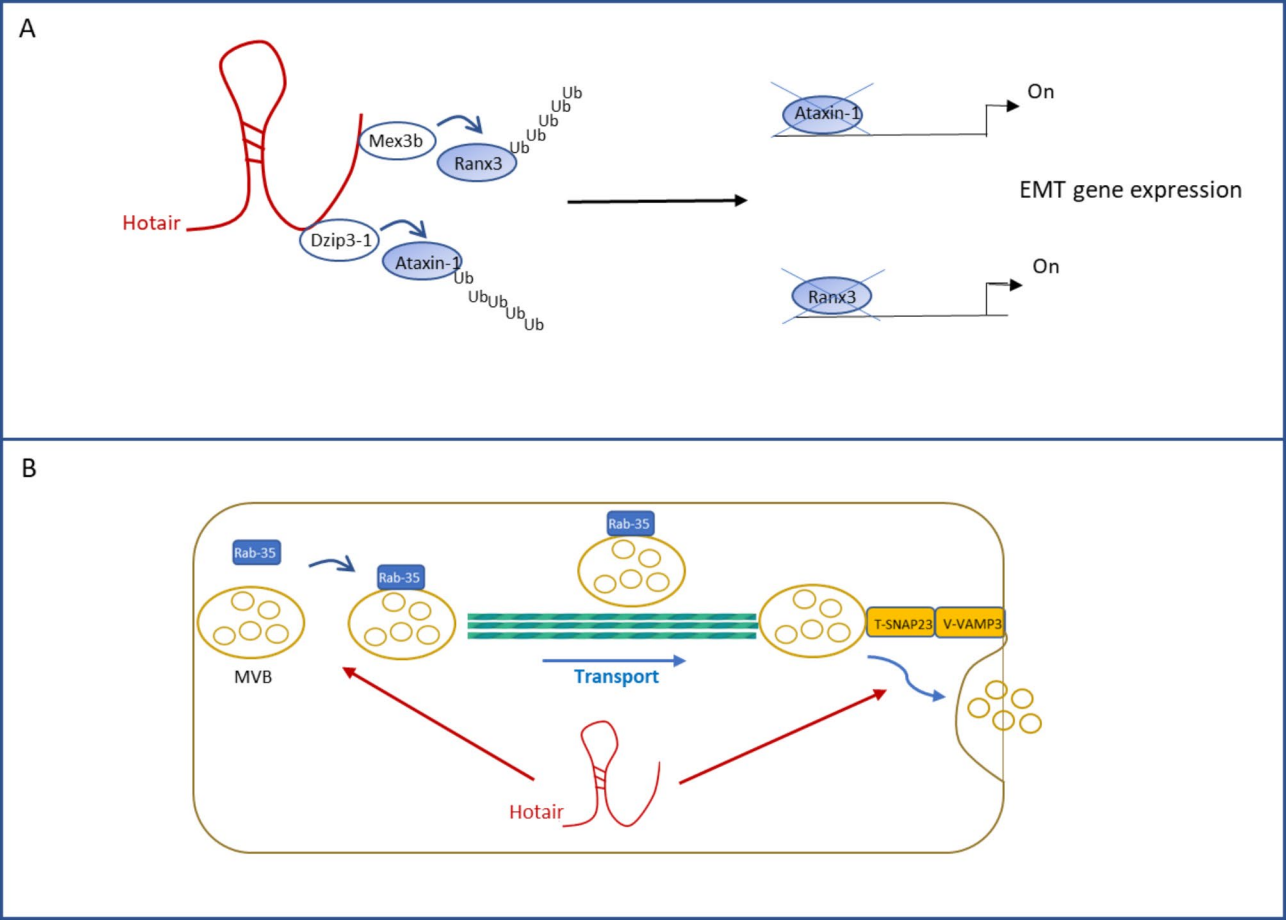
Schemes showing the HOTAIR functions in post-translational regulation of protein activity. **(A)** HOTAIR recruits a molecular platform including the E3 ligases Dzip3 and Mex3b. It can facilitate the assembly of Ataxin-1 with Dzip3, as well as of Runx2 with Mex3b, thus promoting ubiquitination and degradation of the transcription inhibitors and EMT-related gene expression **(B)** HOTAIR regulates MVB transport by controlling the expression and the localization of RAB-GTPase on MVB membrane. Moreover, the lncRNA promotes the docking between V-SNARE and T-SNARE allowing the fusion of MBV and plasma-membrane and the exosome secretion

Trans-acting lncRNAs may modulate the levels and the activities of interacting proteins. In accordance, HOTAIR may affect the ubiquitin–proteasome pathway as an assembly platform for the E3 ubiquitin ligases Dzip3 and Mex3b. Notably, by means of these interactions, HOTAIR facilitates the ubiquitination of different substrates. Particularly, HOTAIR can accelerate the degradation of Ataxin-1 by Dzip3 [62] (Fig. 4A). Ataxin-1 can have a role in gene regulation by its ability to bind to transcriptional repressors in the chromatin [112]. In cervical cancer cells, ataxin-1 binds Snail promoter when the gene is repressed, while its knockdown directly induces Snail expression together with the acquisition of a mesenchymal phenotype [113]. Similarly in gastric cancer cells, by means of the interaction with Mex3b, HOTAIR can induce the ubiquitination, and subsequent degradation, of Runx3, thus enhancing cell invasion capacity [114] (Fig. 4A). Notably, the transcription factor Runx3 represents a key regulator of epithelial cell plasticity *via* regulation of several effectors of EMT and its loss sensitizes the cell to the trans-differentiation [115].

Furthermore, HOTAIR is involved in the control of the release of exosomes by the cell, potentially affecting cell-cell communication in the tumor microenvironment. Starting from the positive correlation in hepatocarcinoma between HOTAIR overexpression and the enrichment of genes involved in exosome secretion, Yang and Coworkers investigated the mechanisms by which HOTAIR influences the exosome release [116] (Fig. 4B). They firstly focused on the HOTAIR-mediated effect on the transport of multivesicular bodies (MVBs) to plasma membrane and found that the lncRNA positively regulates the expression of the Ras-related protein Rab-35 (Rab35), that mediates MVB transport along microtubules. Furthermore, HOTAIR specifically binds Rab35, as demonstrated by RIP and pull-down assays, controlling its localization. Moreover, HOTAIR permits the fusion of MVBs with the membrane by affecting the colocalization of VAMP3 and SNAP23, members of the soluble N-ethylmaleimide-sensitive fusion factor attachment protein receptors (SNARE). SNAP23 receptor was found further regulated by HOTAIR by phosphorylation *via* the mammalian target of rapamycin (mTOR) signaling [116].

### The functional role of HOTAIR epi-modifications

Increasing evidence points to the role of a variety of RNA chemical modifications in affecting gene expression. These epi-modifications confer structural and functional diversity to different types of coding and non-coding transcripts, without alterations of RNA sequence. The addition and the removal of chemical groups can be dynamic and depends on regulatory enzymes that act as “writers’’ (i.e. installers) and “erasers” (i.e. removers), while the “readers’’ are deputed to recognize and bind specific modified nucleotides. Among more than 100 internal modifications of RNA in mammalian cells, the N^6^-methyladenosine (m^6^A) is the most prevalent [117]. Interestingly, m^6^A influences RNA homeostasis and dynamics, from pre-mRNA processing to translational efficiency, but also impacts on epigenetic mechanisms of gene expression regulation. Studies using the genetic knockout of m^6^A-modulating molecular machinery components, indeed, provided evidence that m^6^A modifications contribute to the regulation of the chromatin organization. This can be accomplished, for example, by the recruitment, thanks to the readers, of histone modifying enzymes in the chromatin context, or by regulating the function of specific RNAs [118], including lncRNAs. For example, the role of multiple m^6^A sites in controlling the lncRNA Xist-mediated inactivation of X chromosome has been described [119, 120].

With respect to HOTAIR, the role of m^6^A in the specific control of its function has been recently highlighted by Porman and Colleagues in breast cancer cells [121]. These authors, by means of an enhanced version of the crosslinking and immunoprecipitation assay (eCLIP), mapped multiple m^6^A sites on HOTAIR. Particularly, mutagenesis of the adenosine to uracil demonstrated that the residue A783 is required for HOTAIR function in promoting proliferation and invasion of breast cancer cells. Furthermore, RIP analysis provided evidence that this site, when epi-modified, interacts with the m^6^A reader YTHDC1. Moreover, YTHDC1 levels regulate HOTAIR-induced pro-metastatic effects in cancer cells, as proven by overexpression and knock-down experiments. Further i*n vitro* assays indicated that YTHDC1 mediates the association of HOTAIR with chromatin and it is involved in its mediated gene repression. Therefore, the HOTAIR recruitment on the genome can be dependent on the YTHDC1-mediated recognition of m^6^A epi-modification, even if the mechanism of specific targeting in genomic loci remains unclear [121].

Heterogeneous nuclear ribonucleoproteins (hnRNP) A2/B1 were further identified, by quantitative proteomic analysis and successive validation by western blot analysis as specific interactors of HOTAIR [122]. Meredith and co-workers reported that hnRNPA2/B1 knockdown negatively regulates HOTAIR-dependent invasion of breast cancer cells. Particularly, the B1 isoform was found necessary to enhance a direct RNA–RNA interaction between HOTAIR and a target transcript, impacting on HOTAIR recruitment to the corresponding DNA and triggering gene repression. In fact, the Authors propose a model in which the interaction between the lncRNA and this RNA binding protein (RBP) promotes the binding of HOTAIR to specific nascent transcripts allowing the recruitment of PRC2 and the subsequent trimethylation of H3K27 at the correspondent genomic loci [122, 123]. Notably, while hnRNPA2/B1 is a known regulator of RNA fates (by affecting several processes including mRNA trafficking and splicing [124, 125] and miRNA sorting in extracellular vesicles [126]), this RBP was also characterized as nuclear reader of the m^6^A mark [127]. Further investigation is required to evaluate if there are m^6^A modifications of HOTAIR, excluding the A783 site targeted by YTHDC1 [121], that may be involved in hnRNPA2/B1 interaction. In fact, even if eCLIP analysis reported by Porman et al. [121] indicates that m^6^A modifications on HOTAIR are not directly recognised by hnRNPB1, it cannot be excluded that they can indirectly promote the binding. Interestingly, hnRNPA2/B1 has a role in promoting EMT and activates Snail in hepatocellular and pancreatic cancer progression [128, 129].

Another post-transcriptional modification, conserved in all life domains and linked to translational control, RNA stability and structural functions of different classes of RNA, is the methylation of cytosine (5-methylcytosine, m^5^C) [130]. Notably, site-specific m^5^C could control the ability of RNAs to interact with RNA binding proteins, also in the chromatin context; for example, methylation of Xist affects its ability to bind the PRC2 complex, fundamental to drive the Xist-induced epigenetic gene silencing [131].

RNA bisulfite sequencing approaches in different cancer cell lines demonstrated that HOTAIR is a further m^5^C-methylated lncRNA [131]. By analyzing a short region in proximity of the LSD1-binding site, a C at position 1683 was found invariably methylated in different cell types, despite different levels of expression of HOTAIR. More importantly, the position of this modification suggests that it may affect the ability of HOTAIR to interact with LSD1, required for HOTAIR-induced gene regulation.

### HOTAIR targeting approaches

**Fig. 5:**
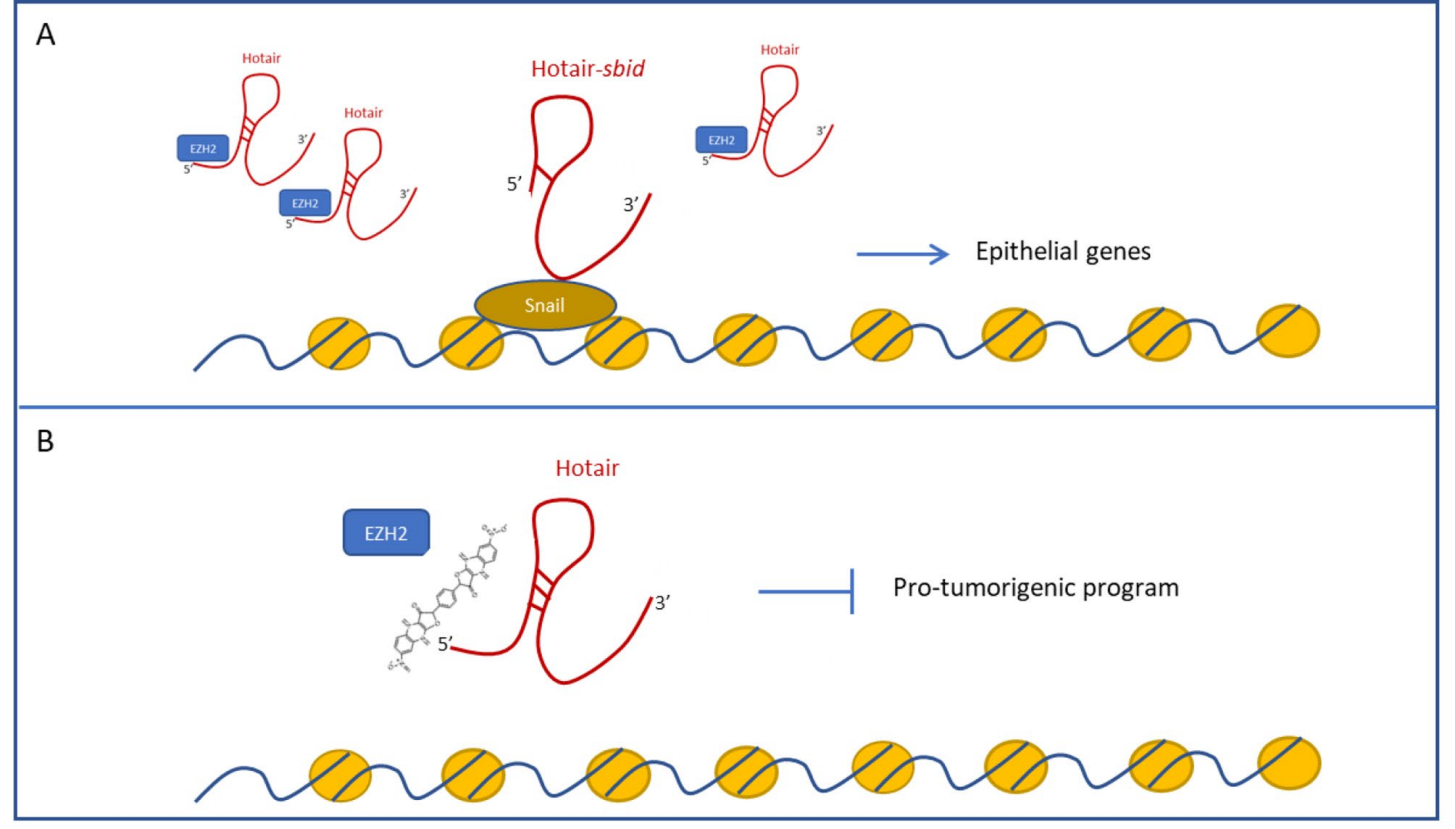
HOTAIR targeting approaches. **(A)** The mutant Hotair-*sbid*, lacking the domain recruiting EZH2, acts as a competitor of the endogenous HOTAIR for Snail binding, allowing the de-repression of epithelial genes. **(B)** The small compound AC1NOD4Q (ADQ) specifically binds the 5’ domain of HOTAIR interfering with the interaction between the lncRNA and EZH2 and with the expression of a pro-tumorigenic program of gene expression

As described above, in the last years HOTAIR has emerged as a master regulator of EMT and its multifaceted role in the control of cell plasticity renders it a suitable target of molecular strategies aimed at impairing the pathological process of the epithelial trans-differentiation. To our knowledge, up today some different approaches to interfere with the HOTAIR function have been proposed (see Fig. 5). A pioneering RNA-based strategy has been recently set to antagonize HOTAIR by designing and in vitro functionally validating a deletion mutant of the lnRNA itself [132]. This mutant, called HOTAIR-*sbid* (for *S*nail *bi*nding *d*omain), does not comprise the EZH2 binding region while still retains the domain for Snail interaction; therefore, it acts as competitor of the endogenous HOTAIR for Snail binding while impairs the assembly of a functional molecular platform, including EZH2, on chromatin (Fig. 5A). As shown, it exhibits a dominant negative function on the HOTAIR-mediated Snail repressive activity on epithelial genes pivotal for the transition, allowing the rescue of a more differentiated phenotype in hepatocarcinoma cells and the protection of non-tumorigenic hepatocytes from the TGF-β1-induced EMT [132].

Advances in the knowledge on structure/function of the HOTAIR/EZH2 complex led to the design of a further approach, aiming at impairing the lncRNA scaffold activity. By means of a high-throughput screening of the PubChem library, Ren and Colleagues identified a small compound, AC1NOD4Q (ADQ), able to specifically bind the 5’ domain of HOTAIR and to interfere with the interaction between the lncRNA and EZH2 (Fig. 5B). Of note, the use of ADQ, by inhibiting H3K27 modifications, impacts on the expression of the HOTAIR target gene nemo-like kinase (NLK). Moreover, ADQ reduces cell migratory activities and, in vivo, the growth and metastasis of xenograft tumors [133].

Furthermore, a general pharmacological approach was proven to control HOTAIR gene expression. Golshan and colleagues, indeed, described that the treatment of breast cancer cells with metformin, a hypoglycemic drug well-known for its anticancer properties [134], promotes the methylation of a CpG-rich sequence located at the downstream region of HOTAIR gene [46] (see Fig. 1A), causing its downregulation [135]. Interestingly, the treatment with metformin was found sufficient to reverse the EMT properties of tumor cells.

## Future challenges and perspectives

The body of evidence reported above confirms that the HOTAIR targeting is a conceivable anti-EMT strategy and future innovative clinical interventions to efficiently counteract epithelial tumor progression should consider these encouraging results [132, 133, 135].

Based on the general relevance of lncRNAs as pleiotropic regulators of cell fate in different pathological contexts, the setting up of approaches to target this class of RNA molecules is a hot topic of current research. Progresses are rapidly attended, considering the growing amount of knowledge of molecular mechanisms of function as well as the advance in structural biology studies. In this scenario, therapeutic RNA molecules are expected to better guarantee the advantage of the sequence specificity, thus limiting off-target effects, even if potential issues could be determined by sequence similarities or overdosing effects. Moreover, they should exhibit low toxicity. However, to date, the productive translation in clinic of RNA-based approaches needs the improvement of two further crucial aspects of the delivery approaches, i.e. the stability of therapeutic molecules and the specificity of the conveyance to target cells (that must be ensured by the vehicle systems, e.g. lipid nanoparticles, exosomes, antibodies or peptides) [136–138]. All these issues should be overcome also for specific strategies aimed at interfering with HOTAIR function. However, a deeper investigation of HOTAIR-binding partners as well as of its chemical modifications are required. In this direction, approaches with small molecules that may target RNA structures or interfere with its interaction with epigenetic modifiers, or other interactors, could also be better pursued [139–141]. Interestingly, with respect to the possible interference with HOTAIR function by the impairment of its epi-modifications, Porman and Colleagues characterized a HOTAIR mutant in the A783 methylated site that lacks the ability to be recruited to the chromatin and to drive the gene expression program of tumor progression in breast cancer cells [121]. These lines of research would better clarify the versatility of HOTAIR functions in cell plasticity and point to the design of conceivably improved strategies.

## Data Availability

Data sharing is not applicable to this article as no datasets were generated or analyzed during the current study.

## Abbreviations

EMT: Epithelial to mesenchymal transition
EMT-TFs: EMT transcriptional factors
ECM: extracellular matrix
MET: mesenchymal to epithelial transition
noncoding RNAs: noncoding RNAs
lncRNAs: ncRNAs; long noncoding RNAs
HOTAIR: HOX transcript antisense intergenic RNA
HOXC: homeobox transcription factor C
HDE: HOXC distal enhancer
3C: forkhead box proteins, FOX proteins
ERs: estrogen receptors
EREs: estrogen response elements
ChIP: chromatin immunoprecipitation
EMSA: electrophoretic mobility shift assay
CBP: CREB-binding protein
TGF-β: transforming growth factor β
HNF4α: nuclear receptor hepatocyte nuclear factor 4-α
CAFs: cancer-associated fibroblasts
NSCLC: non-small cell lung cancer
HuR: human antigen R
PRC2: Polycomb Repressive Complex 2
H3K27me3: histone H3 lysine 27 trimethylation
RIP: RNA immunoprecipitation
ChIRP: co-immunoprecipitation (Co-IP) chromatin isolation by RNA purification
LSD1: lysine specific demethylase 1
H3K4: histone H3 lysine 4
H3K9: histone H3 lysine 9
LOCKs: large organized chromatin K9-modifications domains
ceRNA: competitive endogenous RNA
MMP2: matrix metalloproteinase 2
EVs: extracellular vesicles
MVBs: multivesicular bodies
Rab35: Ras-related protein Rab-35
SNARE: soluble N-ethylmaleimide-sensitive fusion factor attachment protein receptor
mTOR: mammalian target of rapamycin
m^6^A: N^6^-methyladenosine
eCLIP: enhanced version of the crosslinking and immunoprecipitation assay
hnRNPs: heterogeneous nuclear ribonucleoproteins
RBP: RNA binding protein
m^5^C: 5-methylcytosine
HOTAIR-sbid: HOTAIR-*S*nail *bi*nding *d*omain
